# Extent, quality and impact of patient and public involvement in antimicrobial drug development research: A systematic review

**DOI:** 10.1111/hex.12587

**Published:** 2017-07-27

**Authors:** David Evans, Emma Bird, Andy Gibson, Sally Grier, Teh Li Chin, Margaret Stoddart, Alasdair MacGowan, the North Bristol Microbiology Patient Panel, Elizabeth Berry, Richard Campbell, Neil Kane, Royston Marshall, Angelo Micciche, Emily Misselbrook, Roy Smedley

**Affiliations:** ^1^ Department of Health and Social Sciences University of the West of England Bristol UK; ^2^ Department of Infection Sciences Southmead Hospital Bristol UK

**Keywords:** antimicrobial drug development, microbiology, patient and public involvement

## Abstract

**Background:**

Patient and public involvement (PPI) is increasingly recognized as bringing a range of benefits to clinical and health services research. Recent systematic reviews have identified and synthesized many benefits (eg higher recruitment rates) and some costs (eg extra time need). Much of the literature focuses on PPI in long‐term conditions rather than more acute health care in which the majority of microbiological research is undertaken.

**Objectives:**

The aim was to identify the extent, quality and impact of PPI in antimicrobial drug development research. Objectives were to identify any relevant reporting of PPI in antimicrobial research; appraise the quality of reporting on PPI using recognized PPI reporting and critical appraisal tools; and extract and synthesize data on the impact of PPI.

**Search strategy:**

A systematic review was undertaken with a search strategy based on four word groups (PPI, patients, antimicrobial drug development and outcomes). Eight online databases were searched.

**Inclusion criteria:**

English language publication, publication between 1996 and 2016 and studies describing PPI in antimicrobial drug development research.

**Main results:**

No studies were found through online searching that met the search strategy and inclusion criteria. One relevant protocol paper with a brief mention of PPI was identified through expert recommendation. Commentary papers recommending PPI were identified through website searching and expert opinion.

**Discussion and conclusions:**

Despite strong policy guidance encouraging PPI at the international and national levels, and anecdotal accounts of PPI taking place, evidence for the extent, quality and impact of PPI in antimicrobial drug development research has not yet appeared in the peer‐reviewed literature.

## 1 INTRODUCTION

### 1.1 Background

Patient and public involvement (PPI) is increasingly recognized as bringing a range of benefits to health services research. Recent structured^1^ and systematic reviews^2^, ^3^ have identified and synthesized many benefits (eg improved study design, better participant information materials, higher recruitment rates) and some costs and challenges (eg extra time need, risk of tokenism). Public involvement in research has been defined as “an active partnership between the public and researchers in the research process, rather than the use of people as ‘subjects’ of research.”^1^ Much of the literature focuses on PPI in long‐term conditions (eg mental health, rheumatology) rather than more acute and laboratory settings such as those in which much microbiological research is undertaken. There is increasing concern in the field that PPI in research is often either not reported or reported inadequately.^4^ Notably, the bibliography of PPI in research studies published by the UK National Institute for Health Research (NIHR) advisory group INVOLVE^5^ identifies no studies with a focus on PPI in antimicrobial drug development research (ie phase I, II and III studies carried out prior to regulatory agency approval or post‐marketing studies).

Over the last decade, there has been increasing interest in PPI in drug development more generally, with a number of recent calls for action.^6^, ^7^ More in‐depth studies have included Duckenfield and Rangnekar's report on patient group involvement in drug development for chronic and progressive diseases, in particular two case studies on the involvement of patient groups in research on muscular sclerosis and Parkinson's disease.^8^ Houyez discussed patient group involvement in drug development, with particular reference to AIDS patient organizations and activists.^9^ Patient advocacy groups are identified by Smits and Boon as important actors in pharmaceutical innovation.^10^ New PPI forums and initiatives have been established, such as the US Food and Drug Administration (FDA), Patient‐Focused Drug Development initiative^6^ and the European Patients' Academy on Therapeutic Innovation (EUPATI) launched in 2012 as a patient‐led Innovative Medicines Initiative (IMI) project.^11^ EUPATI has published a useful paper of case reports on patient involvement in industry‐led medicines development,^12^ but these all related to drugs for long‐term conditions (including HIV/AIDS) rather than to antimicrobial drug development for acute infections.

This distinction between PPI in long‐term conditions and in antimicrobial drug development is potentially crucial as the opportunities for involvement and impact may vary considerably in the different settings. With many long‐term conditions, there are well‐established international, national and local patient groups that researchers can involve from the beginning of the research journey. In addition, clinical researchers in long‐term conditions may have regular contact over a long period with relevant patients and carers, giving them ample opportunity to involve them early or at any subsequent stage of the research process. By contrast, there are few if any established patient groups for those with bacterial infections. Patients often experience acute bacterial infection as a one‐off experience which is either successfully treated with antibiotics or may be fatal. If recovered, they may thus have less experiential knowledge of their condition. Moreover, such patients are unlikely to have any on‐going identification as an “acute infection” patient, although they may identify with a chronic underlying condition which predisposes to infection, such as cystic fibrosis or diabetes. Thus, the opportunities for involving patients in research on certain types of infection may be limited. What was unknown at the beginning of this study was the extent to which researchers were able to overcome these barriers and successfully involve patients in antimicrobial drug development research. If and when these patients are involved in research, it was also not known whether their impact was similar or different to that reported in previous reviews on the involvement of patients with long‐term conditions in the research process.

As part of IMI‐funded COMBACTE‐MAGNET,^13^ a large programme of research to develop new antimicrobial agents with a strong commitment to developing PPI, there was a requirement to establish the existing evidence base on relevant approaches to PPI that could be built on for the programme. Therefore, this rapid systematic review was conducted to provide an evidence base for the COMBACTE‐MAGNET PPI initiative, in parallel with a rapid qualitative mapping of approaches to PPI in microbiological research which will be reported elsewhere.

### 1.2 Aim and research questions

The aim of the research was to systematically review the microbiology and PPI literature to identify the extent, quality and impact of PPI in antimicrobial drug development research. The research questions were as follows: (i) to what extent is PPI used in antimicrobial drug development research? (ii) in those studies where the use of PPI in antimicrobial research has been reported, what is the quality of reporting? and (iii) in those studies where the use of PPI in antimicrobial research has been reported, what is the impact of PPI?

## 2 METHODS

### 2.1 Design and search strategy

The design and search strategy followed expert guidance^14^ adapted the approach successfully used by one of the authors in a recent systematic review of PPI within surgical research^15^ Four relevant word groups were identified to capture terms relating to PPI, patients, antimicrobial drug development research and outcomes (Table 1). The search strategy sought to reflect the diversity in international terminology relating both to PPI (eg involvement, engagement, participation) and to patients (eg citizens, public, users), but we recognized that at the time there was no consensus on appropriate terminology either in research or in the PPI field more generally. The outcome column relates to impacts on the research rather than to the ultimate health outcomes for patients which are unlikely to be identifiable in PPI research.

**Table 1:** Search terms

PPI	Patients	Antimicrobial drug development	Outcomes
Advisory group^a^	Citizen^a^	Anti‐bacterial	Chang^a^
Patient advocacy	Participant^a^	Antibacterial	Develop^a^
Patient engagement^a^	Patient^a^	Antibiotic^a^	Impact^a^
Patient involvement	Public^a^	Anti‐microbial^a^	Improv^a^
Patient organi^b^ation^a^	User^a^	Antimicrobial^a^	Participant information
Patient participation^a^		Microbiolog^a^	Priorit^a^
Patient and public involvement			Quality Recruit^a^
Patient panel^a^			Research agenda^a^
PPI			Research design^a^
User involvement			Study design^a^

a Indicates truncation.

b Indicates alternative spelling allowed.

The searches were undertaken by a public health researcher (EB), experienced in systematic review methods, under the supervision of the PPI lead (DE). Search terms were applied to titles, abstracts, key words and full texts. A pilot search was performed in MEDLINE to test the search strategy and refine the search terms before the full search of eight databases was undertaken. Databases searched were AMED, ASSIA, CINAHL, Cochrane Database of Systematic Reviews, EMBASE, ISI Web of Knowledge, MEDLINE and PsycINFO. The search strategy was adapted to reflect the structure and parameters of individual databases. Documents retrieved for full text assessment were independently assessed for inclusion by DE to check the validity of inclusion and exclusion decisions. There were no differences in assessment between the two reviewers, but if there had been, it was planned that these should be resolved through discussion.

All original studies of any study design published in English between 1996 and 2016 which described PPI in antimicrobial drug development research were included. Non‐empirical commentary, guidance and opinion pieces were excluded.

In addition, the INVOLVE evidence library was searched, the journals *Health Expectations* and *Research, Involvement and Engagement* manually searched, and references and citations from non‐empirical commentary papers on PPI and antimicrobial research were followed up. The EUPATI and PEW Charitable Trust (work programme on antibiotics) websites were also searched. Experts in the fields of both PPI and antimicrobial research were contacted to identify any relevant papers.

### 2.2 Ethical considerations

Although formal university ethical review was not required as this study did not involve collecting data from human participants, consideration was given as to whether it raised any significant ethical issues. The subject matter (public involvement in research) was not a sensitive issue, and authors of studies reporting microbiological research were unlikely to be vulnerable. PPI in research does not require review and approval, but ethical issues may arise, so ethical considerations as a data extraction field were included.

### 2.3 Quality assessment

Previous reviews have commented on the difficulty of assessing the quality of articles reporting PPI in research.^1^, ^2^ Only a relatively small number of papers report primary research on PPI; more usually, PPI is reported by researchers in relation to studies where the substantive focus is on a health issue, and reporting on the PPI in the primary study is a secondary or subsequent concern. Given that the extensive INVOLVE bibliography and the references included in previous structured and systematic reviews on PPI do not include any studies of PPI in microbiological research,^1^, ^2^, ^3^ it was deemed unlikely that there would be many eligible articles identified in this review, and that it would be unwise to set too demanding a quality threshold. We therefore followed the approach in Brett et al.^2^ and planned to include any study with a clear statement of aims, methods and reported results. It was agreed that eligible studies would be quality appraised for PPI reporting according to an adapted version of the GRIPP checklist^4^ and the critical appraisal guidelines developed by Wright et al.^16^. All included studies were to be independently assessed by EB and DE, and any cases of disagreement were to be resolved through discussion.

### 2.4 Data extraction

Data were to be extracted under the following categories: publication details (author, year, title, journal, volume, number, pages); study details (aims, design, ethics, participants, results); PPI (aims, conceptualization/terminology, number involved, ethical considerations, methods, results, impacts, strengths and limitations identified by authors); and strengths and limitations identified/other comments by reviewer.

### 2.5 Data synthesis

We anticipated that most or all of any reports of PPI in microbiological research would be reflective sections within main study reports, case studies or other forms of qualitative reporting. Thus, a qualitative narrative thematic analysis was planned.^17^ Data from data extraction forms were to have been entered into NVivo 10 and independent analysis identifying key themes relating to the research question undertaken by EB and DE.

### 2.6 Patient and public involvement

As part of the wider literature on the potential benefits and impact of PPI in research, there are an increasing number of published accounts of PPI in systematic review research.^18^, ^19^ In principle, it would have been preferable to have had PPI in this review from the beginning. However, this rapid review was undertaken on a short timescale, while a microbiology patient panel was being established as part of our wider PPI initiative for the COMBACTE‐MAGNET research programme. A pragmatic approach was therefore adopted. Potential panel members were alerted to the review and invited to contribute at the introductory microbiology patient panel meeting in October 2015. To facilitate the patients' ability and confidence to contribute short educational inputs on subjects including research methods were delivered by members of the research group. The main PPI on this review took place during a panel meeting in February 2016 where the limited findings of the review were discussed as a main agenda item, and panel members were invited to contribute to shaping the discussion section. Of the 16 panel members, seven chose to join the subgroup discussing the possible content of the systematic review discussion section. A number of the key points in the discussion section below emerged in this subgroup discussion (eg the recommendations in the penultimate paragraph of the discussion section) The draft paper was then sent to the subgroup to review and ensure that their points had been accurately included.

## 3 RESULTS

**Figure 1:**
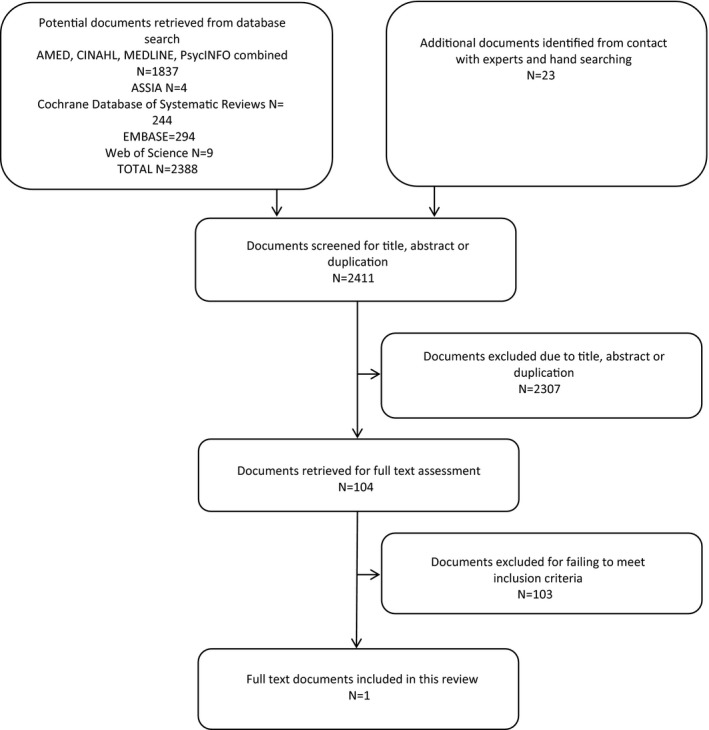
Search results

Database searches identified 2388 potential article titles, with an additional 23 papers identified from contact with experts and hand searching, making a total of 2411 documents where title and abstract were screened for inclusion (Figure 1). After excluding 2307 for duplication or failing to meet inclusion criteria on first screening, 104 were obtained for full text assessment. Of these, 103 were then excluded for failing to meet the inclusion criteria, leaving one^20^ as the only article included in the analysis (Box 1).

Box 1 **Selected article**

1

**Table:** 

Publication details	Thwaites G, Auckland C, Barlow G et al. Adjunctive rifampicin to reduce early mortality from *Staphylococcus aureus* bacteraemia (ARREST): study protocol for a randomised controlled trial. *Trials* 2012; 13:241.
Study details	Protocol for randomized controlled trial to determine whether adjunctive rifampicin reduces all‐cause mortality within 14 d and bacteriological failure or death within 12 wk from randomization.
PPI	Trial developed with the Healthcare‐associated Infection Service Users Research Forum (SURF). A SURF member will represent patients and the public on the Trial Steering Committee. Reports advice on inclusion of incapacitated adults and the application of the Mental Capacity Act, and the information provided to patients. Reports plans for assisting with dissemination to health professionals, patients and the wider public.
Strengths and limitations	Explicit discussion of PPI activity and plans but the brevity of the PPI section makes it difficult to judge the extent, quality or impact of PPI.

The key finding of this review is that there were no papers identified which focused on PPI in antimicrobial drug development research and only one in which there was a brief discussion of PPI, secondary to reporting on the main study. Moreover, this one paper was a protocol rather than a report of findings, and it was concerned with a new use of the drug rather than reporting pre‐authorization or post‐marketing PPI. Thus, compared to other areas of clinical and health services research, there is very little evidence of the extent, quality or impact of PPI in antimicrobial drug development research.

Although a protocol paper, the amount of space allocated to the discussion of PPI (81 words) was similar to that found in papers in other areas of clinical research focused on the substance of a trial rather than specifically on PPI. Given the brevity of the PPI section, it was not appropriate to apply either the GRIPP checklist or the quality appraisal guidelines developed by Wright et al. A GRIPP 2 tool has been developed which offers a choice of checklists between a full version for PPI‐focused studies and an abbreviated version for papers with brief PPI sections, but this has not yet been published.^21^

Due to the brevity of the PPI section in our one identified paper, we also followed up with queries to the lead author of the paper. He reported that eight to ten members of the SURF group were involved in initial discussions of the design of the trial, and one PPI member was recruited to the trial steering committee. She wrote the PPI section of the grant application and has advised and helped throughout the trial. Her role included writing the participant information sheets and when the study experienced some issues around consent; she wrote and undertook a questionnaire survey and a small number of interviews with participants. She has presented with the lead author at a PPI trials conference and will be drafting a specific report on PPI and the consent issues as well as the PPI section of the final report to NIHR (Thwaites, personal communication).

## 4 DISCUSSION

It might be considered that finding only one relevant study briefly reporting PPI in antimicrobial drug development research is a negative and therefore a disappointing finding; however, negative findings of systematic reviews are important to publish as the failure to can lead to serious bias or omissions in the literature.^22^ This apparently negative finding is important in that it highlights a lack of reports of PPI in antimicrobial drug development research compared with some other areas of clinical and health services research. By contrast, there is a literature on PPI in other types of drug development research, in particular relating to drugs for long‐term conditions such as Parkinson's disease, multiple sclerosis and AIDS.^6^, ^7^, ^8^, ^10^ Similarly, there is an increasing body of literature on the contribution of PPI in other types of clinical trials^23^ and in experimental medicine research such as tissue banks.^24^ In principle, there is no reason why the types of PPI undertaken and the contribution it has made in these areas should not be replicated in antimicrobial drug development research.

It is possible that our search missed some other examples of brief reports of PPI in antimicrobial research as such papers would not have included PPI or equivalent terms in the titles, abstracts or key words. However, as we also searched full texts, it is very unlikely that any papers with a substantive focus on PPI have been missed. There is also the possibility that the search terms used were insufficient to identify relevant literature, given the documented inconsistencies in PPI terminology and reporting.^4^, ^16^ For example, our search did not include the term “consumer,” a term identified in a recent article describing the development and testing of a search filter for identifying PPI in health research.^25^ However, the proposed search filter was unpublished and therefore unavailable at the time this review was conducted. Furthermore, consultations with experts in both the fields of PPI and antimicrobial research only uncovered one additional paper, suggesting that few, if any, have been missed.

There are two possible explanations for the findings. First, it may be that researchers have indeed undertaken PPI in antimicrobial drug development research, but have simply not written about it as they have focused their reporting on their substantive study findings and have had little incentive to report on the role of PPI. This may well be the case as some funders, in particular the UK National Institute for Health Research (NIHR), have for some years required researchers to include PPI in their research plans. As NIHR funds some microbiological research, one would expect some PPI would have been undertaken by UK microbiology researchers. Moreover, this finding would be consistent with the lack of reporting of PPI in other areas of health services research; the limited word counts allowed in many peer‐reviewed clinical journals and lack of editorial requirement for such reporting, militates against the reporting of PPI. The second possibility is that (despite the requirements of some funders) few or no antimicrobial drug development researchers have included PPI in their work.

In either case, there would appear to be a lack of attention given to PPI by microbiology researchers. Even if PPI is being undertaken and not reported on, it is only by reporting and sharing examples of good practice and problems encountered (and hopefully overcome) that researchers are able to improve their PPI practice and so gain the maximum benefit from undertaking PPI. As our own patient panel pointed out in discussion, it is arguably unethical to involve people in research and not to report or acknowledge it.

Despite some methodological debates, the overwhelming body of evidence points to PPI bringing a range of benefits to clinical and health services research in general, and drug development research in particular. There has been a flurry of recent articles calling for more patient involvement in drug development,^9^, ^10^ and although not antimicrobial specific, the benefits they argue apply to antimicrobial research. What we do not have is a specific evidence base around PPI in antimicrobial drug development research. But there is no reason to think that the benefits observed more generally in drug development would not also apply in antimicrobial drug development.

The major differences in antimicrobial drug development do not detract from the potential contribution of patients to the research process. It is partly one of recruitment as patients with acute infection do not have established patient groups and do not usually have a long‐term relationship with microbiology researchers; it is also that patients lived experience of their condition may not be as specific to the drug in development as for those patients with long‐term conditions the drugs are directly targeting. Nonetheless, there is similar potential for patients with acute infection to contribute to the prioritization of research (eg around prioritizing interventions to control microbiological resistance) and the design of trials (eg around recruitment and retention).

Recently, a major IMI‐funded study U‐BIOPRED (Unbiased Biomarkers in Prediction of Respiratory Disease Outcomes) reported on its PPI.^26^ Interestingly, the authors note that this article was produced specifically in response to a comment by Catherine Stihler MEP at a European workshop on the need for researchers to communicate the value of meaningful patient involvement in EU projects. They therefore report their experience of patient involvement, but offer up key principles for success of patient involvement in other research: “involve early, involve deeply, have patients feedback on project progress, include patients in dissemination and help patients convey their own story.”^26^ In addition, they provide a comprehensive list of barriers and difficulties for meaningful involvement, and suggestions from their experience on how to overcome these. Taken together, these principles and guidelines provide a template for other drug development researchers. It is also notable that the principles and guidance are very similar to other advice coming from other IMI projects^27^ and more generic PPI reviews.^1^, ^2^, ^3^, ^5^

In discussing the systematic review findings with our microbiology patient panel, a number of potential solutions to the challenges of PPI in antimicrobial research were suggested by the panel members. These suggestions included that where microbiologists have clinical relationships with patients these need to be nurtured and built upon, in particular keeping in touch with patients post‐treatment. Patient panels or advisory groups need to be established where none exist. Such groups need careful planning and skilled facilitation. In particular, the researchers need to be clear on the purposes of the group, what they want to get out of it. Researchers also need to think about the patients' support and training needs. Providing some form of on‐going education or learning can help keep people engaged; however, it is important for this to be led by the questions and learning needs of the group itself. Such education can help the group build up a level of expertize that enables them to contribute. Patient panel members emphasized the need for researchers to make panel members feel comfortable and safe to ask questions or make points; they need to feel there are no “wrong” or “silly” questions.

A recent study found pharmaceutical industry professionals to be positive but uncertain about PPI in medicines research and development.^28^ In the light of the sparse findings of this systematic review, it is likely that microbiologists and other researchers involved in antimicrobial drug development may have similar attitudes and levels of knowledge. Given the importance that funders such as IMI and NIHR now place on PPI, not to mention the ethical imperative expressed by patient groups for PPI to both take place and be reported, and the wealth of examples of the benefits of PPI and guidance from related areas of clinical and health services research on how to do it, the time has surely come for a sustained effort to develop and report PPI in antimicrobial drug development research.

## CONFLICT OF INTEREST

None declared.
